# Reflective insights from developing a palliative care children and young people’s advisory group

**DOI:** 10.1177/0269216320976035

**Published:** 2021-01-12

**Authors:** Anna Roach, Debbie Braybrook, Steve Marshall

**Affiliations:** Florence Nightingale Faculty of Nursing, Midwifery & Palliative Care, Cicely Saunders Institute of Palliative Care, Policy & Rehabilitation, King’s College London, London, UK

**Keywords:** Children, young people, patient and public involvement, young people’s advisory group, palliative care, adolescents

## Abstract

**Background:**

The importance of actively involving patient and public members throughout the different stages of palliative care and health research projects is widely acknowledged, however patient and public involvement work rarely considers insight from children and young people. Although this is becoming increasingly recognised in other areas of research, there is currently no structured guidance on how to best involve children and young people in palliative care research.

**Aim:**

To plan and deliver a Young People’s Advisory Group in palliative care and health research at a secondary school.

**Findings:**

Attending an after-school ‘Health and Social Research Methods Club’ for 11 weeks benefitted children and researchers. Children were taught about data collection methods, data analysis and ethics in health research and used these skills to provide valuable feedback which has been implemented in current palliative care research projects. Children took part in considered discussions around palliative care topics and enjoyed attending the group.

**Conclusion:**

This project has equipped researchers with skills and provided a structured template for future Young People’s Advisory Groups, ensuring the unique voices of children and young people are considered and valued in future palliative care research.

**What is already known about the topic?**

- Patient and public involvement is an important part of the research process.
- Children and young people have a right to be involved in research.
- Young People’s Advisory Groups have been successfully developed in other research areas, educating and encouraging children and young people to be involved in research.

**What this paper adds?**

- Developing a Young People’s Advisory Group benefits both children and young people, and researchers in health and palliative care research.
- Children and young people value involvement in current research projects and enjoy discussing issues in palliative care.
- Structured session plans allow researchers to replicate this group in other settings.

**Implications for practice, theory or policy**

- Palliative care researchers should not avoid involving children and young people for fear of discussing sensitive topics.
- Educating children and young people about palliative care research can equip them with skills to provide valuable feedback to future research projects at varying stages.
- Schools can be an ideal setting for launching a Young People’s Advisory Group, reaching out beyond typical health research groups based in hospitals.

## Background

Patient and public involvement in research is increasingly considered as essential for high-quality research. Public involvement can be defined as involving public members to help identify research priorities, design studies, collect, analyse and interpret data and disseminate research findings, ensuring research remains relevant to the populations in which it is conducted.^1,2^ This also applies to projects concerning children and young people (referred to as children hereafter). Article 12 of the United Nations Convention on the Rights of the Child states that all children capable of forming their own views, have a right to express those views in matters affecting them,^3^ suggesting children can, and should be encouraged to be involved in research. The benefits of children participating throughout the research process are shown to be two-fold: researchers gain further insight after considering different perspectives, and children can be taught new skills, gain confidence and feel empowered.^4,5^ Literature suggests engagement with children can be structured using a participatory tool called the ‘Ladder of Participation’,^6,7^ which ensures researchers deliver meaningful sessions where children can actively participate, avoiding tokenistic involvement.^8,9^ Further literature outlines a pathway which identifies a minimum level of children’s involvement to be achieved to endorse the UN Convention.^10^

The Cicely Saunders Institute of Palliative Care, Policy and Rehabilitation (CSI) was recently awarded funding for two primary research studies investigating what matters to children and families facing serious illness (the C-POS study)^11^ and the needs of children and young people pre-bereavement (the SCYP study).^12^ During the development of study protocols and patient facing materials, researchers looked to external Young People’s Advisory Groups (YPAGs) to provide feedback as although the CSI has an active public involvement strategy group, it is made up of solely adult members. YPAGs are a forum where researchers can engage with children on issues relevant to the ideas, design and dissemination of a project.^13^ Comments from external YPAGs offered unique perspectives and were extremely valuable, therefore CSI researchers sought to develop their own palliative care YPAG. As future palliative care research seeks to engage children and their families, it is increasingly important to develop and involve these groups to ensure that the research remains relevant and accessible.

## Aim

To plan and deliver a young people’s advisory group (YPAG) at a secondary school for 11 weeks.

## Methods

Researchers set up a ‘Health and Social Research Methods’ after-school group at a boys’ secondary school which aimed to educate children about health research methods, encourage thinking around new research ideas and involve children in providing feedback for future studies. The group was led by three researchers who had experience working with children and were actively involved in conducting interviews for palliative care studies involving children. Researchers approached staff from the school who sat on the advisory group for the department’s SCYP project, building on the previous working relationship to develop the YPAG. Teaching staff suggested that ‘palliative care’ was omitted from the group’s name as this may potentially deter children and parents. Researchers agreed and the group was oversubscribed after the first day of being advertised. The final group was made up of 10 male students from different ethnic backgrounds, ranging from 11 to 13 years old.

Researchers were able to learn from the CSI’s robust existing public involvement strategy group who provide valuable project feedback to researchers in the department.^14^ Lessons were also learned from ENRICH,^15^ Involve,^16^ attending an ‘Involving Young People in Research’ masterclass session run by the Centre for Public Engagement^17^ as well as previous experience with existing YPAGs. At all points, researchers sought to achieve a level of child participation that adheres to Article 12 of the UN Convention.

## Findings

The YPAG offered a unique combination of both education and consultation. The project was scheduled to run for 11 weeks and the initial draft programme was flexible and adapted as the group progressed. The session outlines are provided in Table 1. Researchers delivered 5 weeks of teaching followed by a session where the group had the opportunity to devise their own research projects. In the following sessions children were asked to give feedback to visiting researchers and clinicians on different research studies. It was important to teach children about research prior to asking for their feedback: this enabled them to feel equipped to comment on projects they could understand. When planning, delivering, and informally evaluating sessions, researchers reflected on the ‘Ladder of Participation’ tool^7^ to ensure active participation.

**Table 1.** Outline of weekly YPAG sessions.

Session number	Session focus
Week 1	Introductory session
Week 2	Background to social and health research with children
Week 3	Introduction to quantitative research methods
Week 4	Introduction to qualitative research methods
Week 5	Introduction to ethical issues in research
Week 6	Designing own research study
Week 7	Project A Feedback: *Project investigating out of hours palliative care provision*
Week 8	Project B Feedback: *Project developing information for patients and families receiving kidney dialysis*
Week 9	Project C Feedback: *Study exploring culture and pain*
Week 10	Research dissemination: Designing research posters
Week 11	Evaluation session*

* Final session was cancelled due to COVID-19. Researchers sent personalised certificates to the school for all YPAG attendees.

During the qualitative data session (week 4), the YPAG were given pseudonymised quotes from the institute’s SCYP project and discussed themes and the initial coding frame. The group valued the opportunity to input on a current project and were interested in discussing the experiences of children with a parent with a life-limiting illness and palliative diagnosis. Discussions around palliative care also arose in the session exploring ethics in research. The group discussed the importance of avoiding harm and distress when conducting research and the issues surrounding parental consent and confidentiality. The group considered a hypothetical situation about drug trials in terminal illness (as suggested in a resource supporting children to become active researchers^18^). Each child was given a stakeholder perspective to consider, which prompted further discussion around ethical research and specific questions around life-limiting illnesses and palliative care.

When informally evaluating the above programme by asking the YPAG which sessions they particularly enjoyed, they all valued the opportunity to devise their own piece of research, utilising the topics covered in the previous weeks. They were given a structured planning sheet and asked for a specific research question, their data collection methods, analysis plans and any ethical considerations. Individuals presented their project to the YPAG at the end of the session, allowing time for questions and discussions about the feasibility of their research and the methods chosen for data collection and analysis. This session allowed time for both children and researchers to reflect on what had been learnt ahead of providing feedback for the upcoming visiting researcher sessions. Visiting researchers were also asked to complete a feedback form after delivering their session and all indicated that the YPAG feedback was helpful and would be used in grant submissions or later stages of the project.

## Discussion

This project highlights the reciprocal benefits of involving children in health and palliative care research. Children are interested in learning about research methods, being exposed to new topics and enjoy providing feedback, while researchers presenting their projects receive valuable input. The unique perspective from this YPAG has shaped palliative care research projects from the initial development stage and children welcomed the opportunity to play an active role in their development. In most existing health research YPAGs, children are often patients or have a sibling under the care of a hospital or hospice. This group was distinctly different as participants arrived naïve to both research and health education, suggesting feasibility to replicate this group in other schools.

One of the biggest challenges was encouraging research staff to present projects to the YPAG. Researchers voiced fears in making their subject age-appropriate and approaching sensitive topics. The presented projects explored: out of hours palliative care provision, ideas on how to present information for families of patients receiving kidney dialysis and a study exploring culture and pain. Each project was at differing stages of development including pre-funding. The YPAG reviewed and critiqued pre-existing material and provided valuable insight for future dissemination ideas. It should therefore be emphasised that children are willing and are able to engage in all projects, regardless of subject matter.

At the beginning of this project, researchers negotiated the group name with known staff at the school to avoid focus on death and dying. Future YPAGs may benefit from explicitly outlining their focus on palliative care as children showed interest and maturity in talking about potentially distressing topics. However, future YPAGs set up in schools where researchers do not have a pre-existing relationship with staff should seek the school’s advice over the name of the group as they have further insight to the children and families from that school.

As the group was not part of a research study, formal ethical approval was not necessary. A list of after-school clubs was presented to children and their families and parents gave permission for their children to attend the ‘Health and Social Research Methods Club’. When informally asking the group why they chose the YPAG, most were interested as the school had not previously offered a research group and many thought this would look good on their future CV and school applications.

Future groups may also benefit from having a formal evaluation session (in this instance our evaluation session was postponed due to COVID-19 restrictions) to allow children time to discuss their experience of being part of a YPAG and how this could be improved for future groups. Researchers should also consider virtual ways to work with schools to involve and engage children in YPAGs and research following the COVID-19 pandemic which limits face to face contact.

## Conclusion

Developing a YPAG requires extensive preparation and each session requires flexibility. Researchers now have an existing template to build on, edit and share with other teams. As palliative care research continues to place increasing value on hearing the voices of children, it remains important to consider children’s involvement throughout the entire research process, from inception to dissemination. Developing a successful palliative care and health research YPAG has shown benefits for children and researchers from a variety of projects. Researchers from the CSI are now equipped to set up further YPAGs, providing valuable insight for future palliative care research.
